# Effect of Flame Treatment on Bonding Performance of GF/EP Pultrusion Sheets Used for VARI Process

**DOI:** 10.3390/polym15051266

**Published:** 2023-03-02

**Authors:** Yu Zhang, Yundong Ji, Dongfeng Cao, Hongyuan Zhang, Hongda Chen, Haixiao Hu

**Affiliations:** 1 Foshan Xianhu Laboratory of the Advanced Energy Science and Technology Guangdong Laboratory, Foshan 528000, China; 2 State Key Laboratory of Advanced Technology for Materials Synthesis and Processing, Wuhan University of Technology, Wuhan 430070, China; 3 Luoyang Sunrui Rubber & Plastic Science and Technology Co., Ltd., Luoyang 471023, China; 4 Hubei Key Laboratory of Theory and Application of Advanced Materials Mechanics, Wuhan University of Technology, Wuhan 430070, China

**Keywords:** pultrusion process, glass fiber-reinforced epoxy composites, VARI, flame treatment, bonding property

## Abstract

This paper presents an easy and low-cost flame treatment method to improve the bonding performance of GF/EP (Glass Fiber-Reinforced Epoxy) pultrusion plates, which are using widely for large size wind blades. In order to explore the effect of flame treatment on the bonding performance of the precast GF/EP pultruded sheet vs. the infusion plate, the GF/EP pultruded sheets were treated with different flame treatment cycles and were embedded in the fiber fabrics during the vacuum-assisted resin infusion process (VARI). The bonding shear strengths were measured by tensile shear tests. It is found that after 1, 3, 5, and 7 flame treatments, the tensile shear strength between the GF/EP pultrusion plate and infusion plate increased by 8.0%, 13.3%, 22.44%, and −2.1%, respectively. This indicates that the maximum tensile shear strength can be obtained after five times of flame treatment. In addition, DCB and ENF tests were also adopted to characterize the fracture toughness of the bonding interface with the optimal flame treatment. It is found that the optimal treatment gives increments of 21.84% and 78.36% for G I C and G II C, respectively. Finally, the surficial topography of the flame-treated GF/EP pultruded sheets were characterized by optical microscopy, SEM, contact angle test, FTIR, and XPS. The results show that flame treatment plays an impact on the interfacial performance through the combination of physical meshing locking and chemical bonding mechanism. Proper flame treatment would remove the weak boundary layer and mold release agent on the surface of the GF/EP pultruded sheet, etch the bonding surface and improve the oxygen-containing polar groups, such as C–O and O–C=O, to improve the surface roughness and surface tension coefficient of pultruded sheet to enhance the bonding performance. Excessive flame treatment destroys the integrity of epoxy matrix on bonding surface which results into the exposure of the glass fiber, and the carbonization of release agent and resin on the surface loosen the surficial structure, which reduces the bonding properties.

## 1. Introduction

In recent years, with the emergence of the energy crisis and environmental pollution problems, it is generally believed that renewable energy such as wind energy is conducive to achieving carbon dioxide emission reduction, environmental protection, energy conservation, and energy security, so global green energy has developed rapidly [1,2]. As a pollution-free clean energy, wind energy has many advantages, such as large reserves and wide distribution, and wind power generation is also favored by countries because of its rapid development into one of the most representative green energy sources [3,4]. Based on its own national conditions, China has deployed strategies such as “carbon neutrality” and “carbon peaking”, and the wind power industry has developed rapidly with its strategic support [5], In 2021, China contributed 80% of the world’s offshore wind capacity, with a cumulative capacity of 27.7 GW, an impressive growth rate [6,7]. China’s installed wind power capacity has ranked first in the world for 12 consecutive years [8]. According to China’s National Energy Administration, as of the end of March 2022, the cumulative installed capacity of wind power in the country was 337 GW.

Bladed beams, webs, and units are widely used composites in wind turbines. Commonly used reinforcements are glass fiber and carbon fiber, and commonly used resin substrates and adhesives are epoxide and polyurethane. Carbon fiber-reinforced polymer has the advantages of high strength-to-weight ratio and corrosion resistance; thus, it has been used to manufacture crucial components which sustain high loads [9,10,11,12]. However, glass fiber-reinforced polymer is relatively low in price and has better fracture toughness [13,14,15], especially when glass fiber is combined with epoxy resin. Therefore, generally, large wind blades will choose to use glass fiber-reinforced epoxy resin as the largest amount of composite material. The use of large-scale wind turbines is an important path for wind power to reduce costs and increase efficiency, and it is also an established trend in the development of the industry, in order to pursue large-scale development, improve wind power efficiency, and increase the energy conversion rate, new requirements are put forward for the forming process of the blades [16,17].

**Figure 1:**
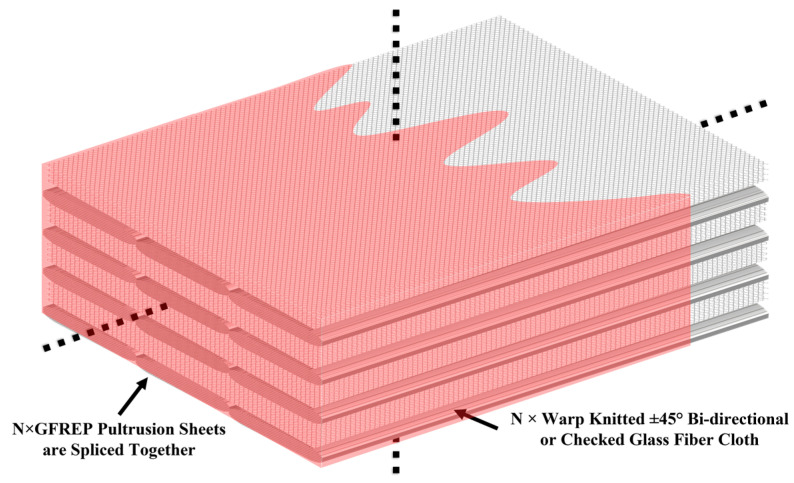
A GF/EP pultrusion sheet is used as a schematic of the girders.

Pultruded fiber-reinforced polymer materials have many outstanding characteristics, such as flexibility, high strength, and chemical resistance, so they have been widely used in many fields, such as construction and infrastructure structures [18,19]. Secondary vacuum injection molding technology of a prefabricated pultrusion beam has been developed. Compared with the traditional blade full size vacuum injection molding process, the prefabricated pultrusion beam secondary vacuum injection molding process uses a large number of pultrusion plates, so it has the advantages of being a simpler process, having a higher material utilization rate, fewer product defects, higher fiber content, with a specific strength, and with a specific modulus [20,21,22,23]. Thus, it is more suitable for making large wind blades. The typical secondary vacuum infusion forming process of prefabricated pultruded girder is to chord splicing and stack several pultruded plates with a thickness of about 5 mm and a width between 100~200 mm to achieve the width and thickness required for blade structure design. During the stacking process, a sandwich glass fiber dry fabric is laid between the pultruded plate layers, and the whole is subjected to secondary vacuum infusion after stacking to act as the main beam cap and other structures of the wind turbine blades [24,25,26,27]. A schematic of the process is shown in Figure 1.

In the secondary infusion molding process of the pultruded girder of the precast blade, the internal release agent (such as INT-1890M) used in the pultrusion process will produce enrichment on the outer surface of the product, which reduces the bonding strength between the pultruded sheet and the secondary infusion layer, thereby restricting the overall performance of the wind blade. At present, in industrial production, it is necessary to add a release cloth to the outer surface of the pultruded board and peel it off before use, so as to remove the surface layer and improve the bonding strength between the pultruded board and the secondary infusion composite layer [28,29,30,31]. However, the increase in release cloth not only increases the complexity of the process, but also significantly increases the manufacturing cost.

At present, a variety of surface treatment methods for the bonding surface performance of bonding materials have been developed [32,33,34], such as mechanical grinding, anodizing treatment, plasma treatment, and flame treatment [35]. Mechanical grinding affects bond strength through mechanical interlocking and adsorption effects, but it is environmentally unfriendly and affects operator health [36,37]. Anodizing increases the bonding strength through mechanical interlocking effect, but as an electrochemical process, it uses a large number of chemical reagents, it is unfriendly to the environment, and it is complex [38,39]. Plasma is the fourth state of matter in which neutral and charged species exist; plasma processing is complex and expensive, and can be completed by mechanical interlocking, while functional groups can be generated to increase bond strength [40,41]. Compared with the above treatment methods, flame treatment is a promising surface treatment method in industrial applications because of its automatic process, faster processing rate, and simple method [42].

Jin Gyu Kim et al. [43] treated aramid/epoxy composite material with a flame and silane coupling agent, measured the single-lap shear strength of the adhesive joint composed of flame-treated aramid/epoxy composite adhesive and epoxy resin adhesive, and characterized the mechanical and chemical interactions between aramid/epoxy composite material and epoxy adhesive. Tomo Takeda et al. [44] investigated the mechanical performance of adhesively bonded carbon fiber-reinforced polymer composite joints prepared with flame-based surface treatment. Adhesively bonded single-lap joint specimens were tested under tensile static loading, and their strength and failure mode were examined. Muhammad Akhsin Muflikhun et al. [45,46] mentioned using interlaminar fracture toughness to characterize the bonding quality of composites.

However, until now, research on the impact of flame treatment on the bonding performance of composites has been characterized by a limited range of methodologies. The majority of studies have focused exclusively on single-lap joint experiments, providing a limited understanding of the overall impact on bonding performance. Furthermore, the utilization of flame treatment on laminated plates produced via pultruded processes has been largely overlooked in the literature. The literature survey provided the essential knowledge needed in identifying some of the key areas investigated in this research program. The research on the surface treatment process of composite pultruded sheet has the real development demand of industrial low cost. This also has academic research value in the field of composite materials.

Therefore, aiming at the interface bonding problem of secondary infusion in the secondary vacuum infusion molding process of a prefabricated GF/EP pultruded girder, this paper explores the effect of flame treatment on the bonding strength between a GF/EP pultruded plate and a GF/EP infusion plate. Using a GF/EP pultruded sheet treated with gradient flame as the base mold, the specimen with close bonding between a GF/EP pultruded plate and infusion plate was prepared by using the VARI process, and the influence of flame treatment on the bonding strength between specimens was explored by tensile shear test; the ideal flame treatment process under the best bonding strength was sought. Furthermore, DCB and ENF tests were carried out on the bonding specimens of the GF/EP pultruded plate and infusion plate under the optimal flame treatment process, and the interlaminar fracture toughness of composite material type I (open type) and type II (slip type) was comprehensively judged, while the physical and chemical mechanisms were explored.

## 2. Materials and Methods

### 2.1. Whole Process of the Experiment

**Figure 2:**
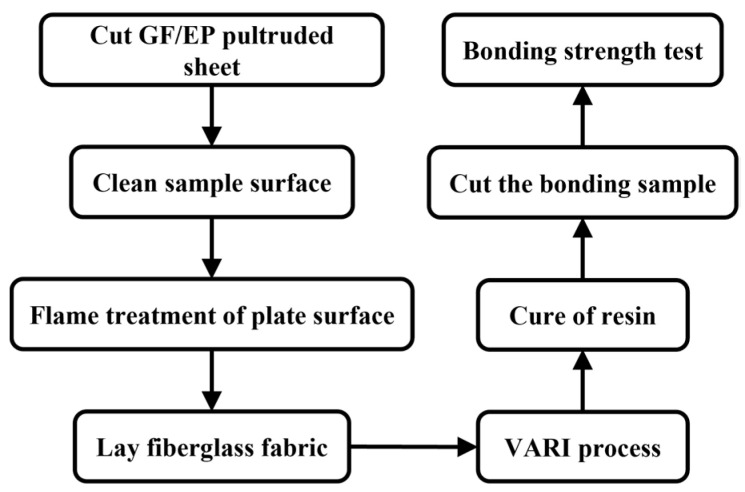
Schematic figure for the sample manufacturing.

**Figure 3:**
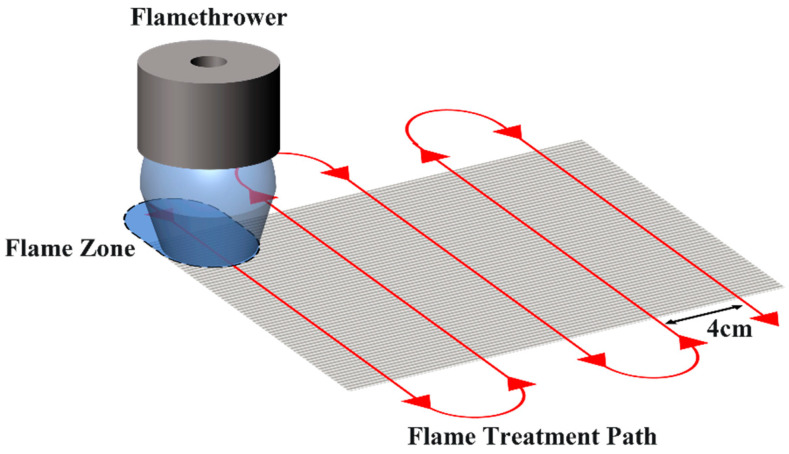
Flame treatment diagram.

Figure 2 is an experimental flowchart including sample preparation. Please refer to the article for details. The GF/EP pultruded sheet (Industrial Grade, Luoyang Shuangrui Rubber and Plastic Technology Co., Ltd., Luoyang, China) used in this paper is made of glass fiber (Owens Corning ws-4000, Shanghai, China), epoxy resin (Shanghai Techstorm 5241A, Shanghai, China), and curing agent (Shanghai Techstorm 5245B, Shanghai, China) by pultruding process with volume fraction of 71 ± 1%. Cut the GF/EP pultruded sheet into 180 mm × 150 mm size, scrub the surface with purified water and dry it, and then wipe it clean with absolute ethanol. After it is completely air-dried, a special butane flamethrower (Based on CAMPSOR 918, Zhejiang CAMAS Outdoor Products Co., Ltd., Zhejiang, China) is used to flame the bonding surface in an attempt to remove the reduction of the bonding strength between the secondary infusion layers by the internal release agent (MOLD WIZ INT-1890M, AXEL Plastics Research Laboratory, Inc., Connecticut, America) in the GF/EP pultrusion process. Under the condition of fixing the medium flame intensity (butane flow rate is 3 L/min), the flamethrower is fixed at the height position where the flame main reaction zone fits the surface of the GF/EP pultruded plate. At this time, the contact area between the flame and the GF/EP pultruded plate as a whole is 16 cm^2^, and the flamethrower is perpendicular to the length direction of the GF/EP pultruded plate, and the reciprocating movement is carried out along the surface at a rate of 15 cm/s to complete the flame treatment of the plate. The flame movement path is shown in Figure 3.

**Figure 4:**
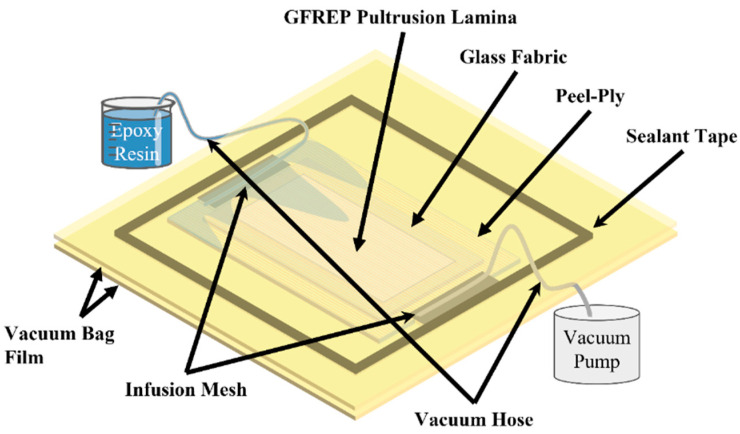
Vacuum-assisted resin infusion diagram.

On the single-layer vacuum bag with the GF/EP plate as the bottom mold, there are multiple layers of 0.35 mm thick glass fiber twill fabric (EKBX240 biaxial seam weave ±45°, Changzhou Putai Glass Fiber Products Co., Ltd., Changzhou, China) on the bonding surface, and introducing epoxy resin (SWANCOR 2511-1A/BS, Shangwei (Tianjin) Wind Power Materials Co., Ltd., Tianjin, China) through the VARI process (as shown in Figure 4), the ratio of the main agent to the curing agent is 100:30. After the vacuum introduction is completed, the entire VARI infusion plate is cured at room temperature for 6 h, which was then put into the oven to cure at 60 °C for 8 h.

During the specimen preparation process, the stiffness of the plates on both sides of the specimen bonding surface is similar by laying different layers of twill glass fiber cloth. After laying five layers of 0.35 mm thick glass fiber twill cloth on the bonding surface, the specimens for tensile shearing were infused. For the preparation of double cantilever beam (DCB) and end notched flexure (ENF) specimens, 6 and 14 layers of 0.35 mm thick glass fiber twill cloth, respectively, were laid on the bonding surface, and a 0.1 mm thick polytetrafluoroethylene film with a pre-fabricated length of 50 mm microcracks was also placed between the GF/EP sheet and one end of the glass fiber cloth. After all the preparations were completed, vacuum infusion was initiated.

**Figure 5:**
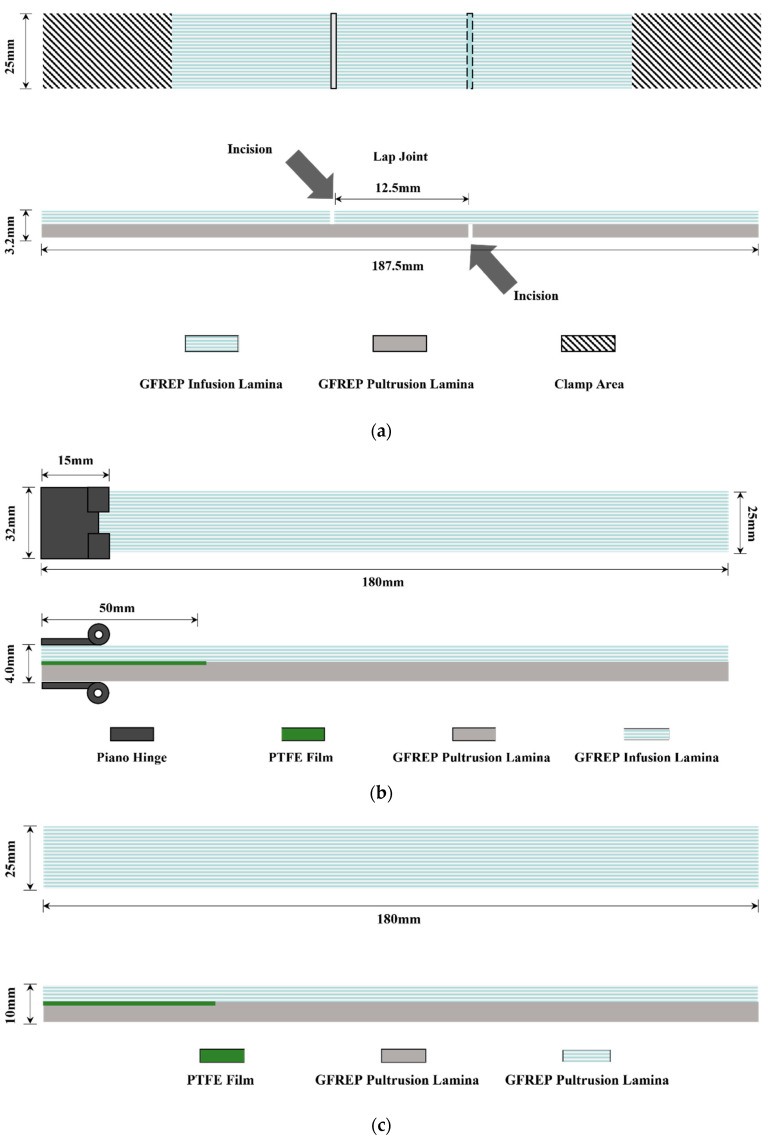
Sample diagrams: (**a**) diagram of the tensile and shear strength; (**b**) diagram of the double cantilever beam; (**c**) diagram of the end-notched flexure.

After curing was completed, the composite plates prepared by VARI were cut into the standard tensile shear specimens, DCB specimens, and ENF specimens shown in Figure 5 according to ASTM D1002-10 [47], ASTM D5528-13 [48,49], and ASTM D7905-2019 [50,51].

### 2.2. Test and Characterization

In this paper, the tensile shear strength obtained by tensile shear test is used to characterize the bonding strength between the GF/EP pultruded plate and infusion plate, so as to study the effect of flame treatment on the bonding effect between the GF/EP pultruded plate and infusion plate, and obtain the best bonding strength and corresponding best flame treatment process by comparing the tensile shear strength data of the specimen after each gradient flame treatment. In order to comprehensively characterize the effect of flame treatment on the bonding performance between the GF/EP pultruded sheet and infusion plate, DCB and ENF tests were also carried out under the optimal flame treatment process to determine the fracture toughness *G_IC_* and *G_IIC_* of the interlaminar opening type and slip type of composite materials.

The tensile shear test is carried out in strict accordance with ASTM D1002-10, and the bonding strength between the GF/EP pultruded sheet and infusion plate was tested after flame 0, 1, 3, 5, and 7 flame treatments by a universal testing machine (UTM5105X, Shenzhen Sansi Zongheng Technology Co., Ltd.), and five effective samples per group were obtained. The load is applied by displacement control, and the loading rate is 1.0 mm/min. Type I interlaminar fracture toughness *G_IC_* and type II fracture toughness *G_ⅡC_* were obtained by DCB and ENF tests of the hydraulic fatigue testing machine (Landmark 370.25, MTS Systems Corporation). The DCB test is performed in strict accordance with ASTM D5528-13, with five valid specimens per group, and the test adopts displacement control to apply the load, while the loading rate is 2.0 mm/min. The ENF test is performed in strict accordance with ASTM D7905-2019, with five valid specimens per group, and the test also uses displacement control to apply a load at a loading rate of 0.5 mm/min. After loading failure, the morphology of the bonding surface was observed by an optical microscope (Zeiss Axiolab 5, Beijing Preses Instrument Co., Ltd., Beijing, China).

The GF/EP pultrusion plate was cut to a size of 1 mm × 1 mm, the surface was cleaned with pure water and wiped clean with alcohol, to be completely dry, while the sample underwent flame treatment 0, 1, 3, 5, and 7 times, and was then immediately subjected to an SEM test. SEM (JSM-7500F, Nippon Electronics Co., Ltd., Huizhou, China) was used to characterize the effect of the flame treatment on the surface morphology of GF/EP pultrusion plates.

**Figure 6:**
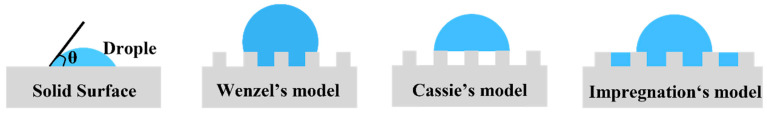
Diagram of the contact angle and wetting model.

When the trace water droplets are on the surface of the clean sample, the angle formed between the tangent line of the gas–liquid interface and the solid–liquid junction line at the intersection of solid, liquid, and gas is called the contact angle. A schematic of the wetting theory model of Young, Wenzel, and Cassie is shown in Figure 6, and the contact angle can be seen as a means of quantifying the surface energy of a solid [52,53].

In this experiment, the contact angle of the plate to water was used as a key index to quantify the wettability of the GF/EP pultruded sheet to epoxy resin, because the contact angle of different sizes reflected the surface tension coefficient of the GF/EP pultruded sheet and water under different flame treatment processes, and the surface tension coefficient is an expression of the polarity content of surface groups, which greatly affects the bonding between the GF/EP pultruded sheet and epoxy resin. The contact angle test of the GF/EP pultruded sheet to water under gradient flame treatment can intuitively characterize the effect of flame treatment on the wettability between the GF/EP pultruded sheet and epoxy resin by semi-quantitative surface tension coefficient. The small contact angle indicates that the plate has good wettability to epoxy resin, which is conducive to the flow of epoxy resin to form a large bonding area and more mechanical interlocking structures.

Therefore, we tested the contact angle (KRUSS-DSA-25E, Krüsh Scientific Instruments (Shanghai) Co., Ltd., Shanghai, China) of GF/EP pultrusion plates before and after gradient flame treatment. In addition, the effects of gradient flame treatment on the surface groups and elements of GF/EP pultrusion plates were studied by XPS (K-Alpha, Thermo Fisher Scientific (China) Co., Ltd., Shanghai, China) and FTIR (Niolet iN10, Thermo Fisher Scientific (China) Co., Ltd.) characterization.

## 3. Results

### 3.1. Bond Strength Data and Analysis

#### 3.1.1. Tensile Shear Strength

**Figure 7:**
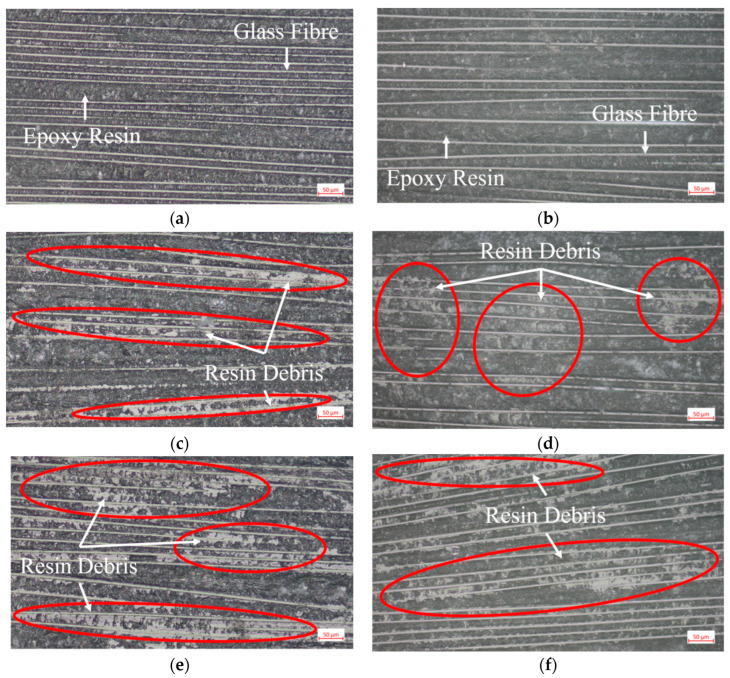

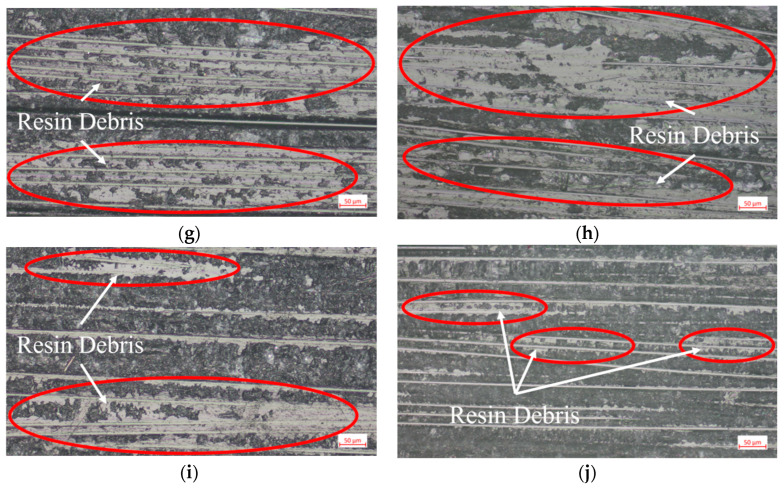
Micrographs of the failure surfaces of the tensile shear specimen with different times of flame treatment: (**a**) GF/EP infusion sheets that have not undergone flame treatment; (**b**) GF/EP pultruded sheet that has not undergone flame treatment; (**c**) GF/EP infusion sheet with one flame treatment; (**d**) GF/EP pultruded sheet after one flame treatment; (**e**) GF/EP infusion sheet after three flame treatments; (**f**) GF/EP pultruded sheet after three flame treatments; (**g**) GF/EP infusion sheet after five flame treatments; (**h**) GF/EP pultruded sheet after five flame treatments; (**i**) GF/EP infusion sheet after seven flame treatments; (**j**) GF/EP pultruded sheet after seven flame treatments.

An optical micrograph of the bonded surface after a gradient flame-treated bonded specimen fails to load is shown in Figure 7.

From Figure 7, it can be seen that the surface of the resin matrix between the glass fibers on the failure bonding surface of the sample without flame treatment is smooth (Figure 7a,b), and the failure form is pure interface failure. With the increase of the number of flame treatments, from 1–5 flame treatments (Figure 7c–h), more and more white resin chips attached to the bonding surface of the GF/EP pultruded plate and infusion plate due to the failure of resin between the fibers during the tensile loading process, the section is rougher, and the failure form changes from pure interface failure to mixed failure of interface failure and resin matrix failure. This phenomenon is significant with the increase of the number of flame treatment, which indicates to a certain extent that the bonding strength between the resin on the bonding surface also increases. After seven flame treatments, there are still some white fragments on the bonding surface of the GF/EP pultruded sheet and its bonded infusion plate (Figure 7i,j), and the failure form is still a mixed failure of interface failure and matrix failure, but compared with the bonding surface of seven flame-treated specimens (Figure 7g,h), the number of white fragments is significantly reduced, which indicates that the bonding strength of the two bonding surfaces after seven flame treatments may be less than the bonding strength of five flame treatments.

**Figure 8:**
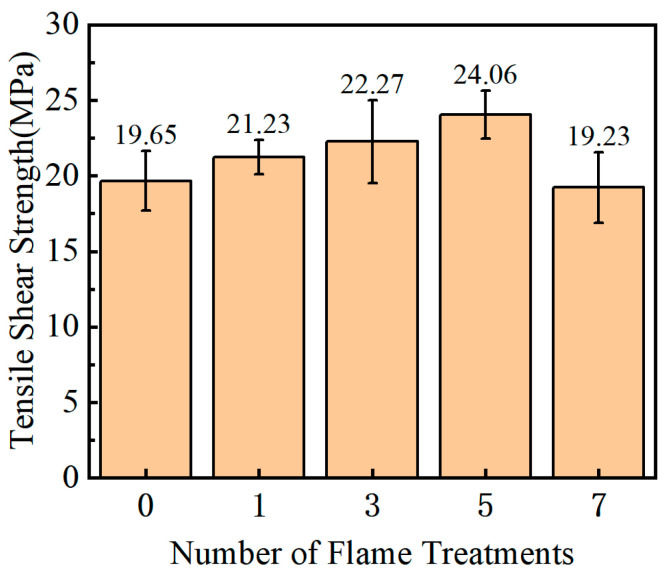
Tensile shear strength.

Figure 8 shows the change in tensile shear strength of a composite adhesive surface with the number of flame treatments.

It can be seen from Figure 8 that the bonding strength first increases and then decreases with the increase of flame treatment times, and reaches a maximum value of 24.06 MPa when flame treatment is performed five times, which is 22.44% higher than when no flame treatment is performed. However, after seven times of flame treatment, the bonding strength decreased, even slightly lower than the bonding strength without flame treatment. Thus, there is an optimal value for the improvement of the bonding strength between the GF/EP pultruded sheet and epoxy resin by flame treatment, and the optimal flame treatment number measured in this experiment is five times.

#### 3.1.2. DCB

**Figure 9:**
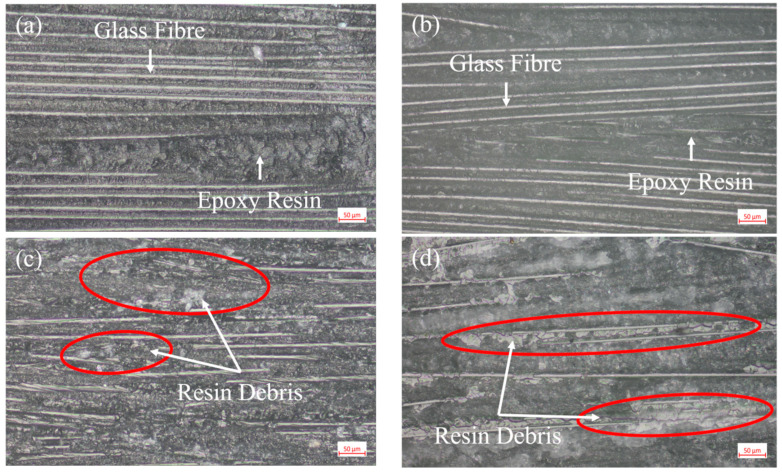
Photomicrograph of the failure surfaces of DCB sample: (**a**) GF/EP infusion sheets that have not undergone flame treatment; (**b**) GF/EP pultruded sheet that has not undergone flame treatment; (**c**) GF/EP infusion sheet after five flame treatments; (**d**) GF/EP pultruded sheet after five flame treatments.

In this part, the influence of the optimal flame treatment process on the failure of the bonding surface of the GF/EP pultruded sheet and infusion plate under the open working condition is explored by using the composites type I interlaminar fracture toughness *G_IC_* obtained by DCB test. An optical microscopic picture of the specimen bonding surface after the DCB test is shown in Figure 9.

From Figure 9, it can be seen that the glass fiber, resin matrix, and interface of the bonding surface of the GF/EP pultrusion sheet without flame treatment are clearly visible, and the resin matrix between the glass fibers is smooth (Figure 9a,b); moreover, the section shows the failure characteristics of pure interface peeling under the DCB open loading condition. After five flame treatments, the GF/EP pultrusion plate bonding surface glass fiber and matrix interface is relatively blurred, the GF/EP plate glass fiber between the glass fiber has scaly resin fragments, the GF/EP bonding surface on the epoxy resin matrix uneven, and the bonding surface is rough (Figure 9c,d), and under DCB open load, the failure form between the two plates can be regarded as both interface failure and resin matrix failure. This difference in the failure morphology of the bonding surface caused by flame treatment can explain to a certain extent why the five flame treatments can improve the bonding between the GF/EP pultruded plate and infusion plate under the open working condition.

**Figure 10:**
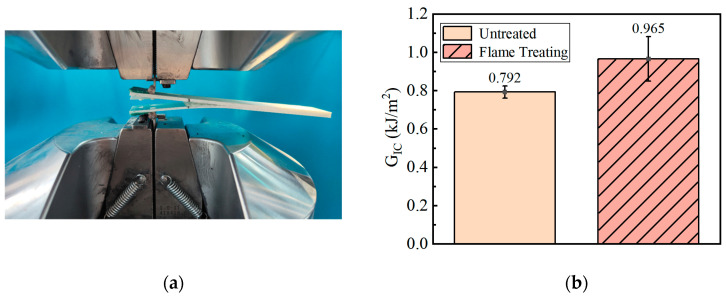
*G_IC_* measurement of the DCB test: (**a**) photos of the experimental process; (**b**) experimental results.

The DCB test data were calculated using the modified beam theory (MBT):

(1) $$G_{IC}=\frac{3P\delta }{2ba}$$

where *P* is the critical load of crack propagation, N; *δ* is the displacement of the loading point, mm; *b* is the width of the specimen, mm; *a* is the length of the layering during the experiment, mm. The calculation results of *G_IC_* are shown in Figure 10.

From the *G_IC_* data in Figure 10, it can be seen that the fracture toughness of the composite material type I between the GF/EP pultruded plate and infusion plate treated by five flame treatment processes is increased by 0.173 kJ/m^2^, which is 21.84% higher than that of the sample without flame treatment. The results show that the five flame treatments can effectively improve the adhesion performance of the secondary infusion interface between the GF/EP pultruded plate and the infusion plate under the open condition.

#### 3.1.3. ENF

**Figure 11:**
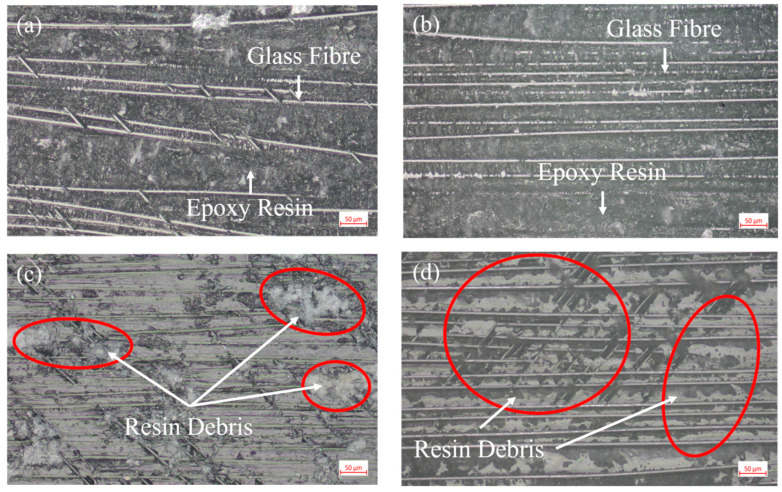
Photomicrographs of the failure surfaces of ENF sample: (**a**) GF/EP infusion sheets that have not undergone flame treatment; (**b**) GF/EP pultruded sheet that has not undergone flame treatment; (**c**) GF/EP infusion sheet after five flame treatments; (**d**) GF/EP pultruded sheet after five flame treatments.

Through the ENF test, the type II fracture toughness *G_IIC_* of the composite was used to characterize the effect of the best flame treatment process on the bonding strength of the GF/EP pultruded sheet and infusion plate under slip condition. An optical micrograph of the specimen bonding surface after the ENF test is shown in Figure 11.

From the comparison of the micromorphology of ENF specimen after failure in Figure 11, it can be seen that compared with the bonding surface of the GF/EP pultruded plate without flame treatment (Figure 11a,b), the bonding surface of the GF/EP pultruded plate after five flame treatments is significantly rougher and the resin matrix fragments attached between the glass fibers are distributed in a large area of continuous blocks, while many resins are left on the bonding surface (Figure 11c,d) after loading and crushing. Similar to the open loading case in Figure 9, the failure of the adhesive layer under the slip condition also changed from pure interface shear failure to a mixed form of interface failure and resin matrix failure, which also indirectly indicates that the anti-slip failure ability of the interface between the GF/EP pultruded sheet and infusion plate was improved after five flame treatments.

**Figure 12:**
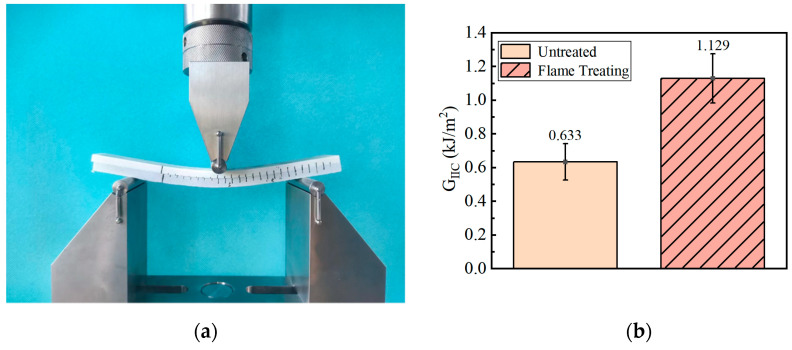
*G_IIC_* measurement of the ENF test: (**a**) photos of the experimental process; (**b**) experimental results.

The ENF test results were calculated using the flexibility calibration method (CC):

(2) $$G_{IIC}=\frac{3mP_{{}_{Max}}^{2}a_{}^{2}}{2B}$$

where *m* is the linear fitting slope of the cubic data of flexibility and crack length, 1/(Nmm^2^); *P* is the critical load of crack propagation, N; *a* is the length of the stratification during the experiment, mm; *B* is the width of the specimen, mm. The results of the *G_IIC_* calculations are shown in Figure 12.

The data of *G_IIC_* in Figure 12 show that the interlaminar fracture toughness of the composite material type II interlaminar fracture of the GF/EP pultruded plate and the bonding surface of the infusion plate treated by five flame treatment processes is increased by 0.496 kJ/m^2^, which is 78.36% higher than that of the overall bonded specimen without flame treatment. This shows that the five flame treatments in this experiment can significantly improve the bonding strength between the GF/EP pultruded plate and infusion plate under the slip condition.

### 3.2. Surface Topography Analysis

**Figure 13:**
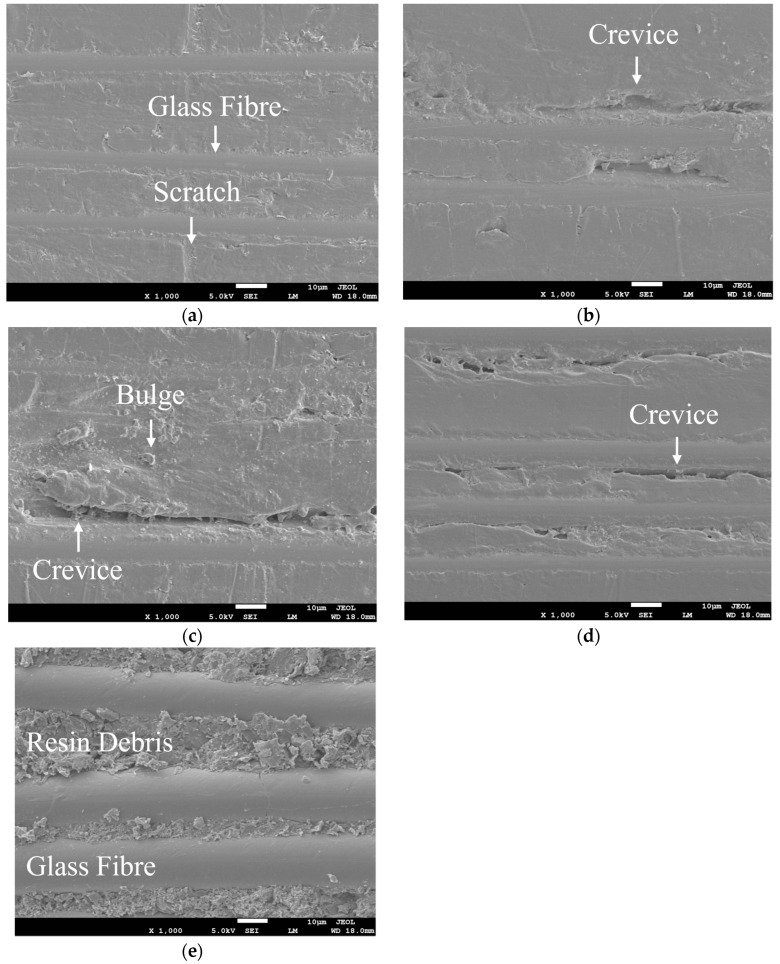
SEM picture of the GF/EP pultruded plate surface: (**a**) no flame treatment; (**b**) one flame treatment; (**c**) three flame treatments; (**d**) five flame treatments; (**e**) seven flame treatments.

In order to explore the influence mechanism of butane flame treatment on the bonding performance of the GF/EP pultruded plate and infusion plate, SEM was used to characterize the surface morphology of the GF/EP pultruded plate without flame treatment and after 1, 3, 5, and 7 gradient flame treatments, respectively, and the results are shown in Figure 13.

Figure 13 shows that the surface of the untreated GF/EP pultruded sheet is smooth, and the fine scratches are caused by wear during production and transport (Figure 13a). After one butane flame treatment, the violent oxidation reaction produces a large amount of heat conduction to the surface of the GF/EP pultruded plate, and the surface epoxy resin is heated and melted, causing it to have a slight debonding from the glass fiber, and local shallow cracks appear at the interface (Figure 13b). After three flame treatments, the epoxy resin is intensified by heat melting, and after cooling, tiny bumps are formed on the surface, and the debonding expands and deepens along the direction of the glass fiber from the local point (Figure 13c). After five flame treatments, a large amount of heat is transferred to the surface of the epoxy resin, the fine scratches and microscopic bumps on the surface all disappear, the interface between the epoxy resin and the glass fiber is obvious, and the interface gap becomes common, continuous, and further deepened (Figure 13d). After seven flame treatments, the epoxy resin on the surface of the GF/EP pultrusion sheet melted a large amount, and after cooling and crystallization, a large number of fine resin chips were formed stacked between the glass fibers, while the cracks between the glass fibers and epoxy resin all disappeared, leaving the glass fibers exposed in a large area (Figure 13e).

It can be seen from the SEM results that moderate flame treatment can melt the epoxy resin on the surface of the GF/EP pultruded plate, and form cracks and bulges after cooling. Through the wetting model and evolution of Wenzel and Cassie [53,54,55,56], it can be seen that the influence of flame treatment improves the surface roughness of the GF/EP pultruded sheet, increases the bonding area between the GF/EP pultrusion surface and epoxy resin, and makes the epoxy resin better infiltrate the surface of the GF/EP pultruded sheet, thereby mechanically interlocking strength. However, when the flame treatment is excessive, the damage caused to the surface of the plate is not conducive to the improvement of bonding strength. For example, after seven flame treatments. A large amount of epoxy resin on the surface of the GF/EP pultrusion sheet is melted and cooled into tiny fragments piled up next to a large area of exposed glass fiber, forming a loose surface structure, and the glass fiber itself is smooth and the surface energy is low. This reduces the mechanical interlocking effect between the GF/EP pultrusion sheet and epoxy resin, resulting in the reduction of bond strength between the two plates.

**Figure 14:**
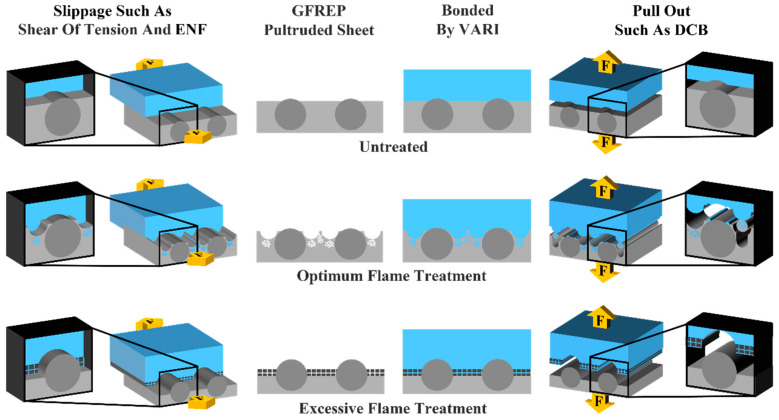
Schematic diagram of the whole process of flame treatment, infusion, and experimental loading on the surface of the GF/EP pultrusion sheet.

By characterizing the surface micromorphology of the GF/EP pultruded sheet after gradient flame treatment, analyzing the morphology of the bonding surface after tensile shear, DCB, and ENF experimental loading failure, combined with tensile shear data, DCB and ENF experimental results, the mechanical locking, and bonding failure of the bonding process of the GF/EP pultruded plate and infusion plate in this paper are schematically illustrated Figure 14 under tensile shear experiment and shear working conditions represented by ENF and pull-out condition represented by DCB.

The surface of the GF/EP pultruded sheet without flame treatment is smooth; the epoxy resin only solidifies on the surface of the plate to form a bond during infusion, and only fails at the interface when loading is damaged. Under the optimal flame treatment process, the EP on the surface of the GF/EP pultrusion plate cracks due to cracking and melting due to heat, and epoxy penetrates into these fine cracks during infusion, forming a firm mechanical lock after solidification, and the loading failure is the mixing failure of the interface and the matrix. The surface resin damage of the GF/EP pultruded sheet with excessive flame treatment is serious, and epoxy penetrates into the damaged resin and bonds with it during infusion, so the bonding strength is low.

The improvement of bonding effect between the GF/EP pultruded plate and infusion plate varies with different working conditions. The improvement effect of ENF (*G_IIC_* increased by 78.36%) is better than that of DCB (*G_IC_* increased by 21.84%), because the epoxy resin bonding between the two plates is not the same under different loading conditions. Under the open type load of DCB, the meshing and locking epoxy resin is subjected to tensile loading. In the slip test of ENF, the epoxy resin with meshing lock is subjected to similar impact loading, and the impact strength (140 MPa) of the epoxy resin used for infusion in this experiment is higher than the tensile strength (67 MPa).

### 3.3. Contact Angle Test

**Figure 15:**
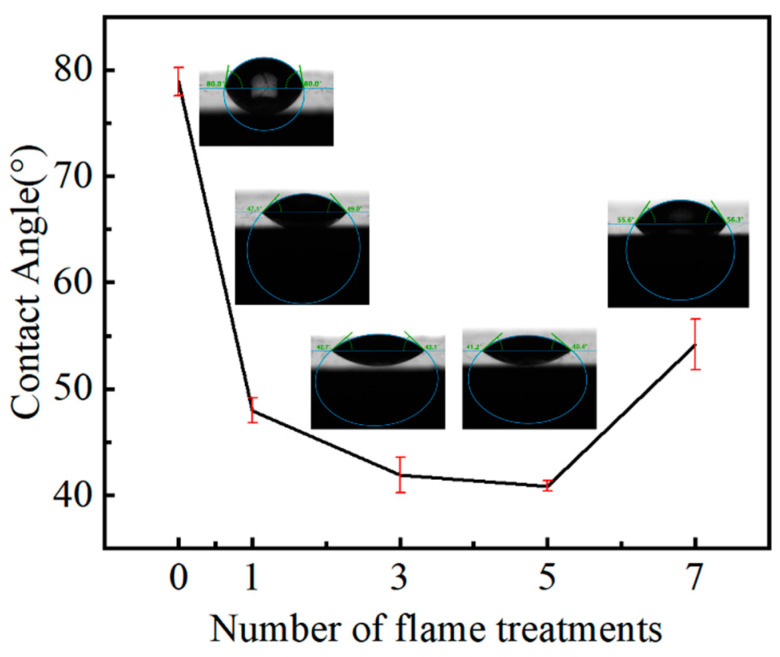
Contact angle test.

The influence of different times of flame treatment on the surface tension coefficient of the GF/EP pultruded sheet was explored by the seat drop contact angle test, and the results are shown in Figure 15.

Figure 15 shows that the flame treatment has a great influence on the contact angle of the GF/EP pultruded plate, and the contact angle is greatly reduced after one flame treatment, but with the increase of the number of flame treatment, the contact angle does not increase linearly and then decreases; it shows a “V” shape change of first falling and then rising. Compared with the contact angle of the GF/EP pultruded sheet surface to water without flame treatment, the contact angle of the GF/EP pultruded plate surface after flame treatment was 48.08°, 41.9°, 40.8°, and 55.95°, which decreased by 39.94%, 47.63%, 49.00%, and 30.06%, respectively. It is worth noting that except for the slight increase of the surface contact angle of the GF/EP pultruded plate after seven flame treatments, the contact angle of the other flame treatment plates decreases with the increase of flame treatment times, which is consistent with the change trend of tensile shear strength. This sudden increase in contact angle may be due to the destruction of the resin on the surface of the plate and the change of surface oxygen groups caused by the destruction of the resin on the surface of the plate and the change of the surface oxygen-containing group due to the exposure of a large area of glass fiber.

### 3.4. FTIR and XPS Analysis

**Figure 16:**
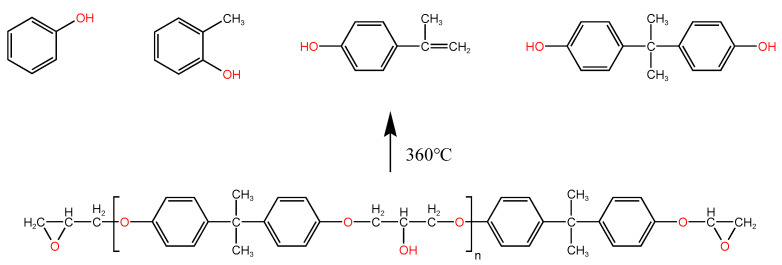
Schematic diagram of a thermal decomposition of EP.

In order to explore the deep cause of this effect, the chemical elements and groups on the surface of pultruded sheet after flame treatment were characterized and analyzed by combining FTIR and XPS. The external flame temperature of the butane flame in this experiment is above 1300 °C, and the internal flame temperature exceeds 500 °C, which is higher than the thermal decomposition temperature of DGEBA (bisphenol A type epoxy resin) in the GF/EP pultruded plate of this experiment of about 360 °C [57,58]. Under this high-temperature treatment, DGEBA is very prone to thermal decomposition, and many scholars have studied the thermal decomposition of DGEBA in detail [59,60]. A typical DGEBA thermal decomposition product is shown in Figure 16.

DGEBA may decompose at about 360 °C to produce phenol, (o-cresol), 2-(benzofur-5-yl)-2-(p-hydroxyphenyl) propane, bisphenol A, and other substances [59,61]. The effects of these substances and their oxides may need to be considered when analyzing FTIR and XPS test results.

#### 3.4.1. FTIR Analysis

**Figure 17:**
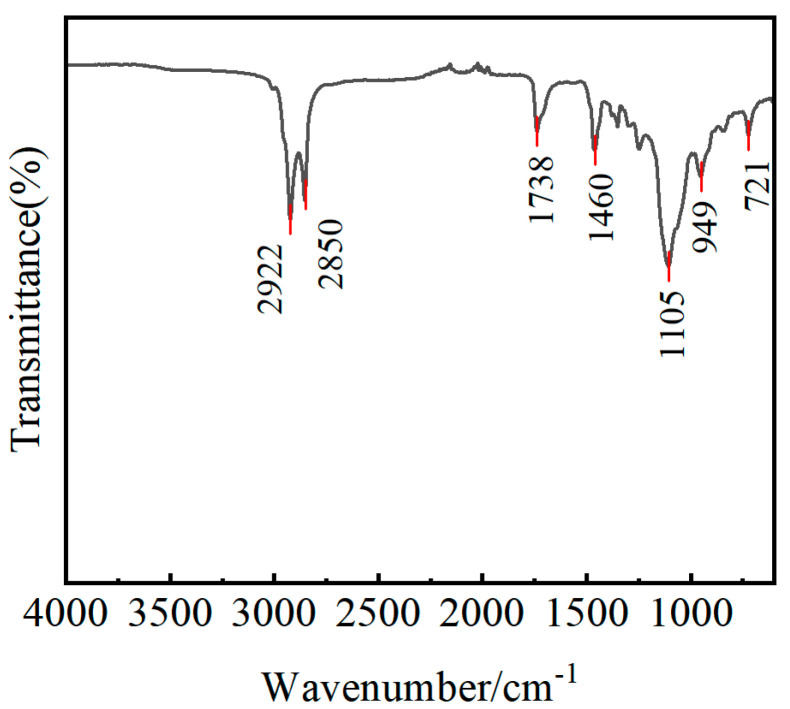
FTIR spectrum of release agent INT−1890M.

**Figure 18:**
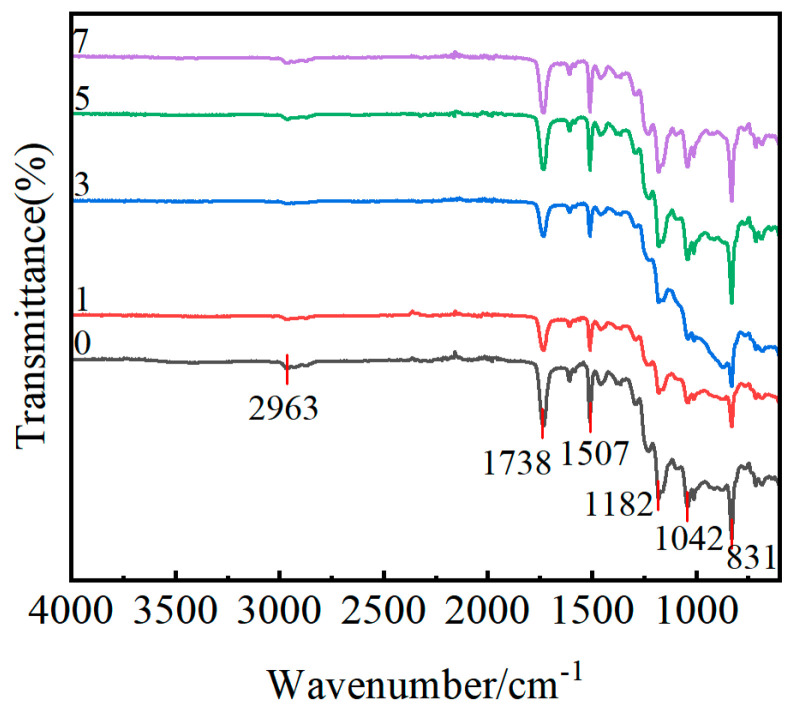
FTIR spectra of samples treated with a different number of flame treatments (0, 1, 3, 5 and 7).

In order to explore the effect of flame treatment on the chemical elements and groups on the surface of the GF/EP pultruded plate, FTIR tests were carried out on the surface of the release agent INT-1890M and the gradient flame-treated the GF/EP pultruded sheet, and the results are shown in Figure 17 and Figure 18.

During the analysis, the spectrum can be divided into two parts: the functional groups region (4000–1333 cm^−1^), which is used to identify the presence and absence of functional groups, and the complex fingerprint region (1333–667 cm^−1^), in which the absorption is less conspicuous [62]. From the infrared spectrum of the release agent INT−1890M, the CH_2_ telescopic vibration absorption peaks are 2922 cm^−1^ and 2853 cm^−1^, the C=O telescopic vibration absorption peak is 1736 cm^−1^, the CH_2_ bending vibration absorption peak is 1460 cm^−1^, the C−O telescopic vibration absorption peak is 1105 cm^−1^, the C−OH off-plane bending absorption peak is 949 cm^−1^, and a CH_2_ out-of-plane deformation absorption peak at 722 cm^−1^ can be clearly seen. This is consistent with the composition of INT−1890M organic fatty acids, esters, and humectants. The resin matrix used in the GF/EP pultrusion sheet is acid anhydride-cured DGEBA, and the vibration absorption peak of methylene (C−H) can be seen at 2962 cm^−1^, the ester carbonyl (C=O) telescopic vibration absorption peak at 1737 cm^−1^, the vibration absorption peak of benzene ring skeleton around 1506 cm^−1^, and the expansion vibration absorption peak of aliphatic ether and aromatic ether bond (C−O−C) at around 1182 cm^−1^ and 1041 cm^−1^. Around 830 cm^−1^, the C−H bending vibration absorption peak can be seen on the multi-substituted benzene ring [63,64,65]. The FTIR spectra of the GF/EP pultruded plates by the gradient flame treatment process show that the position and number of the absorption peaks of each bond have not changed, except for some changes in intensity, which indicates that the gradient flame treatment has changed the content of some substances but has not introduced new chemical bonds.

Compared with the sample without flame treatment, one flame treatment caused the FTIR spectrum of the GF/EP pultruded plate to move down as a whole, which may be because the high-energy flame broke the original equilibrium state of the molecules on the surface of the plate, so that its molecular conformation changed and entered another new equilibrium state. After three flame treatments, the characteristic peaks of the benzene ring at about 1506 cm^−1^ in the FTIR spectra of the plate did not change, and the peaks of 1182 cm^−1^, 1200 cm^−1^, and 830 cm^−1^ in the fingerprint region were enhanced, which may be due to the decomposition of the release agent in the plate and the removal of the benzene ring side group in DGEBA. The FTIR spectra of the plates after five flame treatments showed the enhancement of the 1506 cm^−1^ and 1737 cm^−1^ peaks caused by epoxy backbone rupture and end group oxidation. The FTIR spectral fingerprint area decreased after seven flame treatments, possibly due to the flame further damaging the DGEBA backbone and oxidation of part of the benzene ring opening.

Based on the FTIR results alone, it is impossible to obtain reliable conclusions about the element content, rich morphology, and polar group content of the GF/EP pultruded sheet surface by flame treatment, and further chemical element analysis on the surface of flame-treated plate is required by XPS.

#### 3.4.2. XPS Analysis

**Figure 19:**
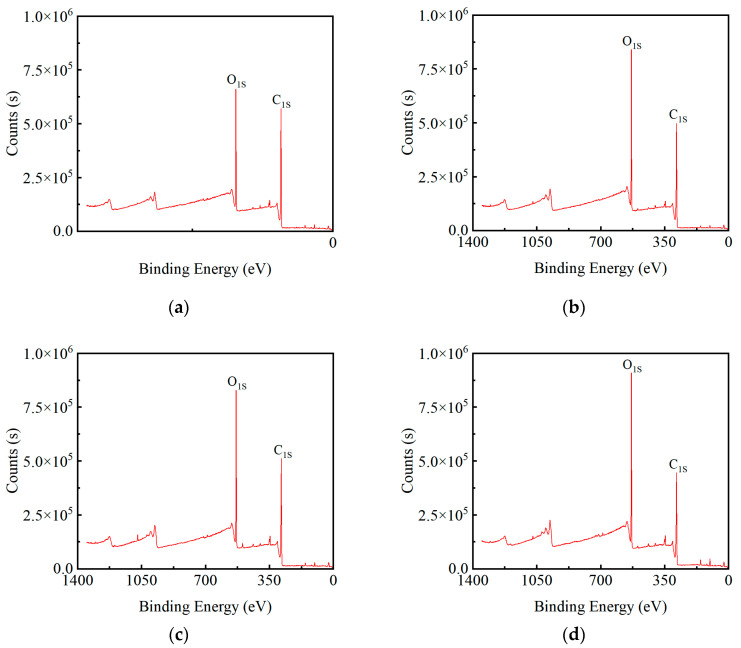

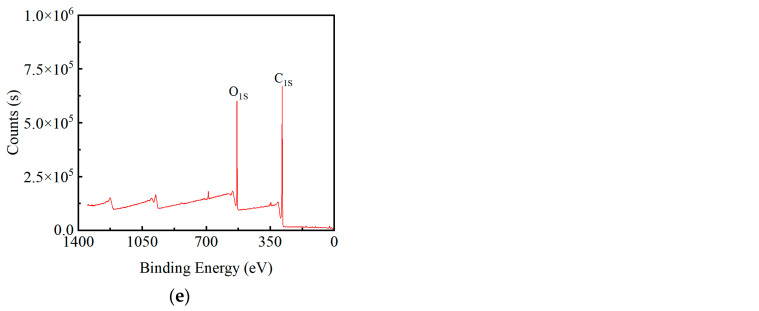
XPS spectra of samples treated with a different number of flame treatments: (**a**) no flame treatment; (**b**) one flame treatment; (**c**) three flame treatments; (**d**) five flame treatments; (**e**) seven flame treatments.

Because of the difference in detection depth, XPS is a more sensitive surface inspection technology than FTIR. The content of carbon, oxygen, and their groups on the surface of the GF/EP pultruded plate after gradient flame treatment was tested by XPS, and the full spectrum of XPS is shown in Figure 19.

The C_1S_ peak of 283.5 eV and the O_1S_ peak height of 530.5 eV in Figure 19 represent the carbon and oxygen content, respectively. It can be seen from the figure that the surface of the GF/EP pultruded plate without flame treatment itself contains a certain amount of oxygen and carbon (Figure 19a). After one butane flame treatment, the surface oxygen content increased and the carbon content decreased, which may be due to the oxidation caused by the violent combustion of butane and oxygen and the removal of some carbon-containing small molecules in the weak boundary layer (Figure 19b). With the increase of the number of flame treatments, the oxygen content was further increased, and the carbon content was further decreased (Figure 19c,d). However, after seven flame treatments, the carbon content increases and is higher than the oxygen content, and the oxygen content is even lower than the sample without flame treatment (Figure 19e), which may be due to excessive flame treatment caused by the release agent in the GF/EP pultrusion plate and resin forming a carbonization layer covering the surface of the plate, which may also be one of the reasons why the contact angle increases after seven flame treatments. The oxygen content in the XPS test was consistent with the tensile shear strength and contact angle test with the change trend of the number of flame treatments.

**Figure 20:**
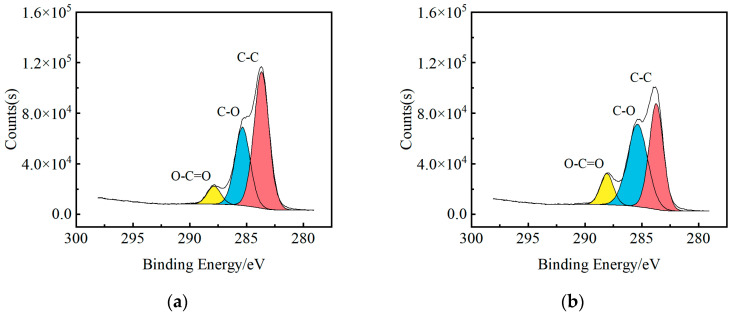

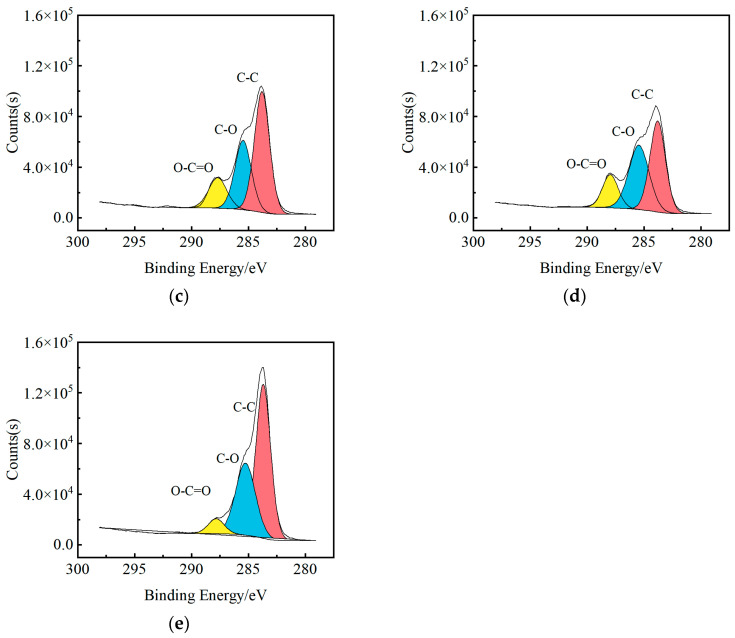
C_1S_ fitting spectra of XPS on the surface of samples treated with a different number of flame treatments: (**a**) no flame treatments; (**b**) one flame treatment; (**c**) three flame treatments; (**d**) five flame treatments; (**e**) seven flame treatments.

To further explore the type and content variation of active groups introduced into the specimen surface by butane flame treatment, a C_1S_ high-resolution scan of the flame-treated specimen surface was performed and the results were fitted, as shown in Figure 20.

**Figure 21:**
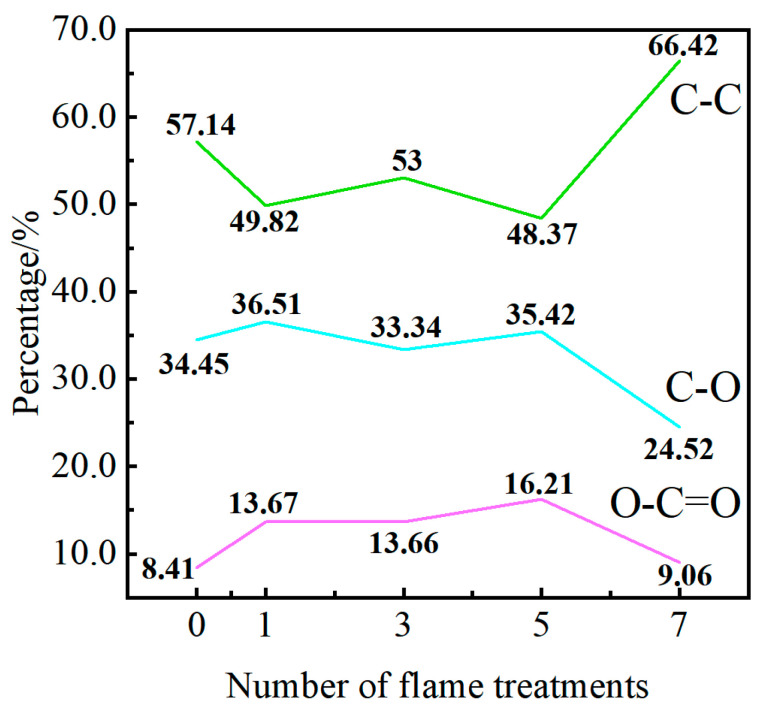
C_1S_ peak fitting results before and after surface treatment.

The C_1S_ spectra of XPS test on the surface of the GF/EP pultruded plate specimens can be seen as fitting the C–C peak of 284.8 eV, the C-O peak of 286.5 eV, and the O–C=O peak of 288.5 eV, while the content of each group is positively correlated with the area between its peak and the background baseline. The change of the three groups with the increase of the number of flame treatment is obtained by Avantage software, as shown in Figure 21.

Combined with FTIR and XPS data analysis, compared with the untreated specimen, the weak boundary layer of the GF/EP pultruded sheet after one flame treatment was removed, and the surface was slightly oxidized, resulting in a decrease in C–C content and an increase in C–O and O–C=O oxygen-containing groups. The release agent in the GF/EP pultruded sheet after three flame treatments was removed, which slightly reduced the content of oxygen-containing groups such as C–O and O–C=O. After further oxidation by five flame treatments, the content of C–O and O–C=O oxygen-containing groups increased and the content of C–C decreased due to the fracture of the epoxy backbone and the oxidation of the end groups of the chain segments, which was conducive to the formation of a strong chemical bond between the GF/EP pultruded plate and infusion plate. The carbon content of the sample after seven flame treatments increased rapidly due to the carbonization of the mold release agent and epoxy resin, resulting in a rapid decrease in the content of C–O and O–C=O oxygen-containing groups.

This study presents a rapid and efficient method to enhance the bonding strength of the GF/EP pultruded sheets, which provides a feasible and cost-effective approach for the prefabricated pultruded beams in wind turbine blades. It is confirmed that the proper flame treatment can improve the bonding strength of composite materials by altering the surface roughness and chemical functional groups, which is consistent with the findings by other researchers. To assess the bonding performance of the GF/EP pultruded sheets comprehensively, the interlaminar fracture toughness characterized by DCB and ENF tests were also used here accompanying with the commonly used tensile shear strength. Due to the limitation of time, further investigations are required to explore certain aspects, such as the effects of different fuels on flame treatment, bonding performance under more complex loading conditions, and comprehensive optimization of the flame treatment process parameters.

## 4. Conclusions

In this study, GF/EP pultrusion plates were treated with a gradient flame and co-bonded to GF/EP infusion plates during the VARI process. The influence of flame treatment on the bonding properties of GF/EP pultrusion plates was evaluated by tensile shear, ENF, and DCB tests and the mechanism of this influence was investigated using various characterization methods.

From the main research conducted here, the following conclusions can be drawn:

(1) The effect of flame treatment on the bonding properties of the GF/EP pultruded sheets was found to be nonlinear. Tensile shear strength initially increased as the number of flame treatments increased in the present study. However, after reaching a maximum value after the fifth treatment, the strength subsequently decreased. The maximum strength value represented an increase in bonding strength of 22.44%.
(2) The *G_IC_* and *G_IIC_* of the bonding line between the GF/EP pultruded and infused sheets prepared using the optimal flame treatment process were found to increase by 21.84% and 78.36%, respectively. This can be attributed to the different modes of stress that the resin, responsible for adhesion, is subjected to during the DCB and ENF tests. The DCB test involves tensile stress, while the ENF test sustains shear stress.
(3) Flame treatment enhances the bonding performance of the GF/EP pultruded plate by removing the weak boundary layer and release agent on the surface of the plate, increasing the roughness of the etched surface, and introducing polar groups containing oxygen.
(4) The excessive flame treatment reduces the bonding performance of the GF/EP pultrusion plate because the excessive flame treatment destroyed the surface of the plate, and is unable to form a firm mechanical lock and carbonized part of the release agent and epoxy resin.

This study provides a low-cost, efficient, and feasible method for improving the bonding strength of GF/EP pultrusion plates, which is widely used in large-diameter wind blades.

## Data Availability

Not applicable.
